# Aerobic exercise training and gut microbiome-associated metabolic shifts in women with overweight: a multi-omic study

**DOI:** 10.1038/s41598-023-38357-6

**Published:** 2023-07-11

**Authors:** Jukka E. Hintikka, Juha P. Ahtiainen, Perttu Permi, Sirpa Jalkanen, Marko Lehtonen, Satu Pekkala

**Affiliations:** 1 Faculty of Sport and Health Sciences, University of Jyväskylä, Jyväskylä, Finland; 2 Department of Biological and Environmental Science, Nanoscience Center, University of Jyväskylä, Jyväskylä, Finland; 3 Department of Chemistry, Nanoscience Center, University of Jyväskylä, Jyväskylä, Finland; 4 Institute of Biotechnology, Helsinki Institute of Life Science, University of Helsinki, Helsinki, Finland; 5 MediCity and InFLAMES Flagship, University of Turku, Turku, Finland; 6 Institute of Biomedicine, University of Turku, Turku, Finland; 7 Faculty of Health Sciences, School of Pharmacy, University of Eastern Finland, Kuopio, Finland

**Keywords:** Biomarkers, Molecular medicine, Lipids, Metabolomics, Metagenomics, Microbiome, Disease prevention, Weight management, Obesity

## Abstract

Physical activity is essential in weight management, improves overall health, and mitigates obesity-related risk markers. Besides inducing changes in systemic metabolism, habitual exercise may improve gut’s microbial diversity and increase the abundance of beneficial taxa in a correlated fashion. Since there is a lack of integrative omics studies on exercise and overweight populations, we studied the metabolomes and gut microbiota associated with programmed exercise in obese individuals. We measured the serum and fecal metabolites of 17 adult women with overweight during a 6-week endurance exercise program. Further, we integrated the exercise-responsive metabolites with variations in the gut microbiome and cardiorespiratory parameters. We found clear correlation with several serum and fecal metabolites, and metabolic pathways, during the exercise period in comparison to the control period, indicating increased lipid oxidation and oxidative stress. Especially, exercise caused co-occurring increase in levels of serum lyso-phosphatidylcholine moieties and fecal glycerophosphocholine. This signature was associated with several microbial metagenome pathways and the abundance of *Akkermansia.* The study demonstrates that, in the absence of body composition changes, aerobic exercise can induce metabolic shifts that provide substrates for beneficial gut microbiota in overweight individuals.

## Introduction

Physical activity in its various forms is essential in weight management. Habitual exercise can improve overall health and mitigate obesity-related risk markers such as insulin resistance, inflammation, and dyslipidemia^1^. Even in the absence of corresponding weight loss, physical activity can lower disease risk and improve overall fitness^2^. The alterations in energy balance and systemic metabolism that occur in response to acute exercise are well characterized and documented^3, 4^. However, within the scope of public health and sports medicine, long-term physical activity and active lifestyle are often of great interest, and their effects on wellbeing and risk factors need more clarification^1, 5^. Well-conducted experimental settings can elucidate the physiological mechanisms behind exercise-induced health benefits, but more studies are needed^6^.

An acute bout of exercise does not only affect systemic metabolism but can also induce transient changes in the composition and metabolism of the gut microbiome^7^. More importantly, increase in habitual physical activity can translate to enhanced microbial diversity and leverage health-beneficial taxa. Consequently, better cardiorespiratory fitness often associates with higher microbial diversity and the abundance of certain exercise-responsive microbial taxa as well^8–10^. The gut microbiome contributes to health and disease by producing bioactive compounds such as short-chain fatty acids, trimethylamine oxide, and amino acid derivatives^11^. These microbes also utilize a lot of endogenous compounds such as bile acids, amino acids, and lactate^11^. Recent studies in mice also indicated specific pathways through which the microbiome-derived metabolites affect motivation to exercise^12^.

Untargeted metabolomics, sometimes also referred to as global metabolomics^13^ or metabolic fingerprinting^14^, aims to characterize large proportions of low-molecular-weight compounds, or metabolites, in a sample matrix in a hypothesis-free manner. As evidenced by the increasing number of studies and new scientific initiatives^13, 15, 16^, this approach is a powerful method for exploring the effects of physical activity in a biological system. The metabolome of a given biological matrix is the function of its genes, transcripts, proteins, and external perturbations; however, the impact of the microbiome is often overlooked in metabolomics studies. This is particularly true for the fecal metabolome, which closely portrays the functions of our gut microbiome^17^. Studies using high-coverage, high-sensitivity metabolomics methods in exercise science are rather scarce^13, 15, 16^, and to our knowledge, there are no experimental multi-omic studies on individuals with overweight.

We have previously shown that sedentary overweight women improved their cardiorespiratory fitness after six weeks of endurance exercise while having shifts in the gut metagenome and microbial composition^18^. A particularly promising effect of exercise was the increase in the bacterial genus *Akkermansia*. The sole member of this genus, *A. muciniphila* has been shown to reduce obesity and insulin resistance, for instance^19^. However, we found no major changes in systemic metabolism in response to exercise as assessed by standard clinical variables and nuclear magnetic resonance (NMR) spectroscopy for targeted plasma metabolites and lipoprotein subclasses^18^. To expand the understanding of the exercise-responsive metabolites, in this study we used a liquid chromatography high resolution mass spectrometry technique (UPLC-HRMS) to characterize the metabolomes in the aforementioned serum and fecal samples. To understand the interplay of systemic and microbial metabolism and their effects on exercise responsiveness, we integrated the metabolic changes with gut microbiome and cardiorespiratory parameters as well as other biochemical variables.

## Results

**Figure 1:**
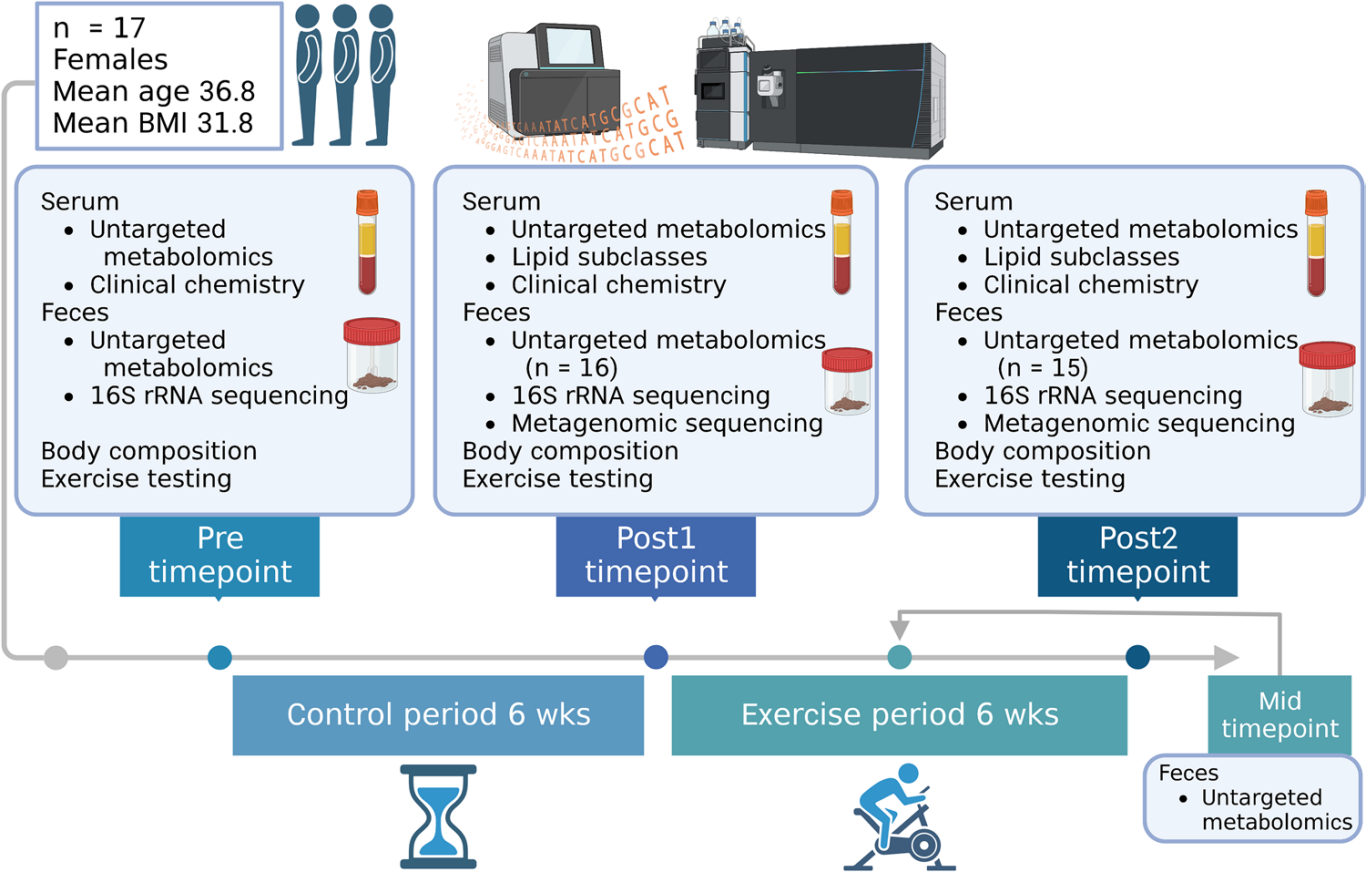
Study design, participants, collected samples and analyses. Created with Biorender.com.

The participants enrolled in a six-week control period and a subsequent six-week exercise period with weekly training sessions (Fig. 1). Body composition and cardiovascular fitness were assessed at three time points i.e., before the control period (pre), after the control period (post1), and after the exercise period (post2). Blood and fecal samples were collected at each time point, and an additional fecal sample was collected at week 4 of the exercise period (mid). We used a high resolution mass spectrometry technique^20^ to characterize the metabolites in the serum and fecal samples from above three time points. Further, using correlative analyses and a network algorithm, we integrated the metabolic changes with gut microbial taxa, metagenomic functions and anthropometric variables to assess the associations between systemic and microbial metabolism.

The main results of the 6-weeks endurance exercise program have been described in detail before^18^. Briefly, the cardiorespiratory fitness of the participants increased, as apparent by increased power and peak oxygen uptake. Android fat mass decreased and the diameter of the vastus lateralis muscle increased. Phospholipids and cholesterol in large very low-density lipoprotein (L-VLDL) particles decreased in plasma and the activity of vascular adhesion protein-1 (VAP-1) decreased in serum. Abundances of the gut microbial phylum Pseudomonadota (former Proteobacteria) and the genus *Akkermansia* decreased and increased, respectively, while several metabolic genes of the gut microbiota were downregulated in response to exercise^18^. In addition, the intakes of nutrients and foods that are known to affect the gut microbiota composition and functions (i.e., carbohydrates, fibre, bread, other grain products, vegetables, fruits, berries, meat, fish, fermented milk products and cheeses) were assessed by a 3-day food record. Besides a slight increase in the proportion of energy from starch, no changes in dietary factors were observed.

### Serum phosphatidylcholines increased during the exercise period

**Figure 2:**
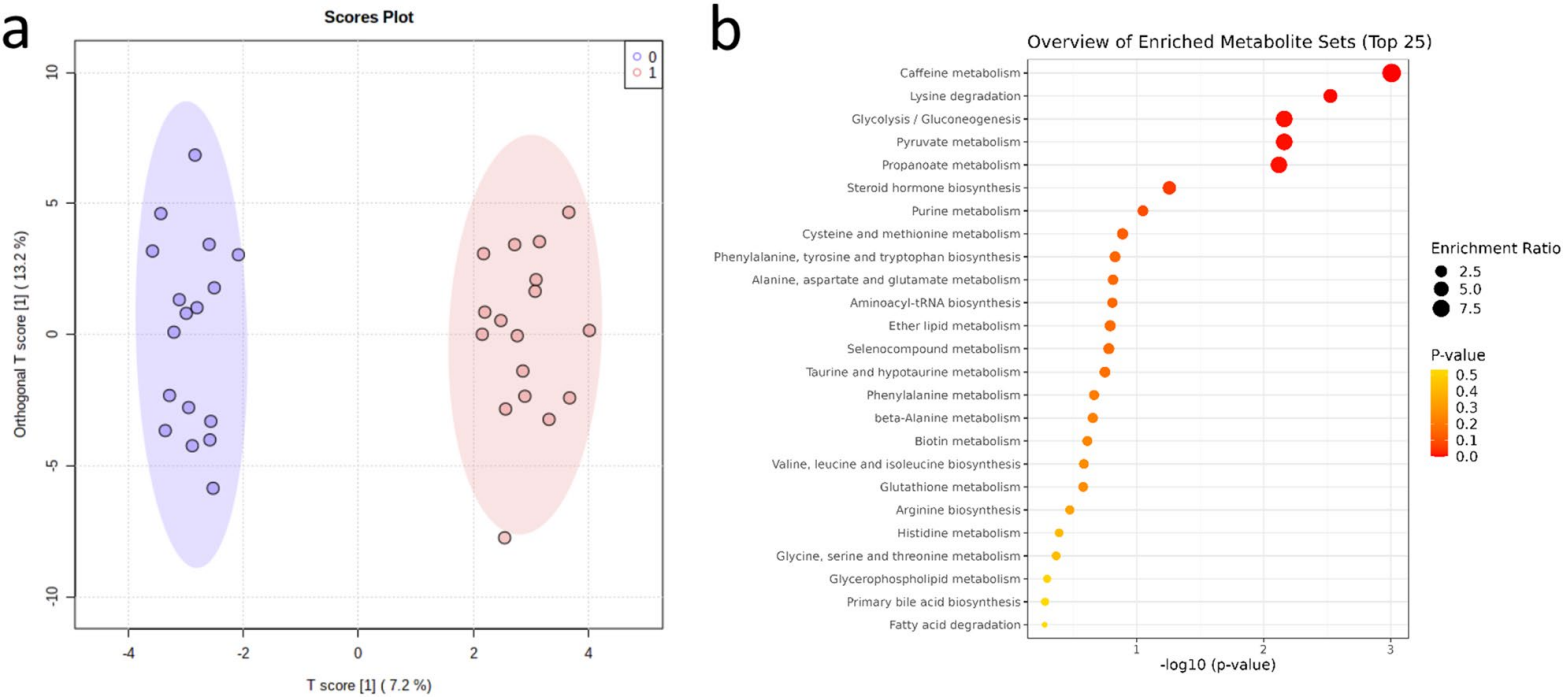
Multivariate metabolome analysis of the serum samples. (**a**) Orthogonal partial least square discriminant analysis (OPLS-DA) of the serum samples (n = 17), with the changes during the control period (0), and the exercise period (1). The model was validated with permutation tests (one hundred permutations p < 0.01) and cross-validation (R2Y = 0.975 and Q2 = 0.637). (**b**) KEGG metabolic pathways in the enrichment analysis of serum samples in MetaboAnalyst. Only caffeine metabolism reached significance after correction for multiple tests.

We identified 124 metabolites in serum. We calculated fold changes for each metabolite during the control and exercise periods and ran multivariate analysis using the orthogonal partial least square discriminant analysis (OPLS-DA) in Metaboanalyst. The details for each serum metabolite, including identification information and multivariate analysis results, are listed in Supplementary Table S1. In OPLS-DA, 13.2% of the variation in the data was explained by the orthogonal component, i.e., interpersonal variation (Fig. 2a). Sixty-two metabolites of interest were selected using the thresholds of p(corr)[1]  < − 0.2 or > 0.2 or VIP-value > 1.0. The cross-validation presented values of R^2^ = 0.975 and Q^2^ = 0.637, and the robustness of this model was measured by 100 permutations tests with p < 0.01. Within metabolic pathways, assessed using the enrichment analysis in Metaboanalyst, caffeine metabolism, lysine degradation, glycolysis, pyruvate metabolism, and propanoate metabolism were significantly enriched, but only caffeine metabolism remained statistically significant after multiple tests correction (Fig. 2b). The disease signatures were also assessed using the enrichment analysis. Interestingly, metabolites affected by exercise were enriched due to alterations in lactate, alanine and purines, and the signature for asthma was found enriched due to alterations in coffee-derived xanthines. However, the disease signatures were not significant after correction for multiple testing (Supplementary Fig. S4).

Twelve metabolites were found to vary during the study, with a notable proportion of phosphatidylcholines (PCs), mainly lyso-phosphatidylcholines (lysoPCs), increasing only during the exercise period (Table 1). Adenosine and caffeine were found to decrease to a half, whereas docosahexaenoic acid 22:6 (DHA), phenylalanylisoleucine, lysoPC(17:0), lysoPC(15:0), lysoPC(16:0), lysoPC(16:1), lysoPC(18:0), PC(18:0_20:4), N6,N6,N6-trimethyllysine (TML) and taurine increased during the exercise period. During the control period, DHA and TML decreased, and caffeine increased. No changes in coffee intake were observed during either the control or the exercise period (p values 0.2 and 0.9, respectively, from repeated measures t-test).

**Table 1:** Mean abundances ± standard deviations of serum metabolites with significant changes during the study.

Compound name	Class/ontology	ANOVA/Friedman q-value	Pre	Post1	Post2
Adenosine	Purine	0.043	4.74 ± 0.93	4.90 ± 0.80	4.44 ± 0.71**
Caffeine	Exposome	0.050	6.99 ± 0.60	**7.22 ± 0.50**^**#**^	6.96 ± 0.64**
Docosahexaenoic acid 22:6^a^	Fatty acid	0.001	6.35 ± 0.20	**6.09 ± 0.21**^**##**^	**6.19 ± 0.25**^**#**^
Phenylalanylisoleucine^a^	Amino acid	0.031	5.23 ± 0.45	5.33 ± 0.38	**5.65 ± 0.38**^#^**
LysoPC(17:0)	Phospholipid	0.007	6.02 ± 0.13	6.03 ± 0.12	**6.09 ± 0.12**^#^**
LysoPC(15:0)^a^	Phospholipid	0.016	5.92 ± 0.12	5.94 ± 0.13	**6.02 ± 0.10**^##^*
LysoPC(16:0)^a^	Phospholipid	0.016	7.01 ± 0.08	7.01 ± 0.08	**7.06 ± 0.07**^##^**
LysoPC(16:1)^a^	Phospholipid	0.072	6.54 ± 0.09	6.51 ± 0.07	6.56 ± 0.09*
LysoPC(18:0)	Phospholipid	0.016	6.28 ± 0.10	6.29 ± 0.10	**6.34 ± 0.10**^##^*
N6,N6,N6-Trimethyllysine	Amino acid	0.007	5.73 ± 0.13	**5.65 ± 0.10**^**##**^	5.73 ± 0.13*
PC(18:0_20:4)^a^	Phospholipid	0.049	6.09 ± 0.26	6.02 ± 0.21	6.27 ± 0.28**
Taurine	Coenzyme	0.016	6.16 ± 0.10	6.17 ± 0.14	**6.23 ± 0.10**^#^**

Values are log10-transformed LC–MS peak signals.

*Pre* before the control period, *Post1* after the control period, *Post2* after the exercise period.

^a^Non-parametric test used. Values in bold indicate a significant change from a previous timepoint. Change from post1 p-value *< 0.05; **< 0.01. Change from pre p-value ^#^< 0.05; ^##^< 0.01.

### The fecal metabolome showed alterations in glycerophospholipids and amino acids

**Figure 3:**
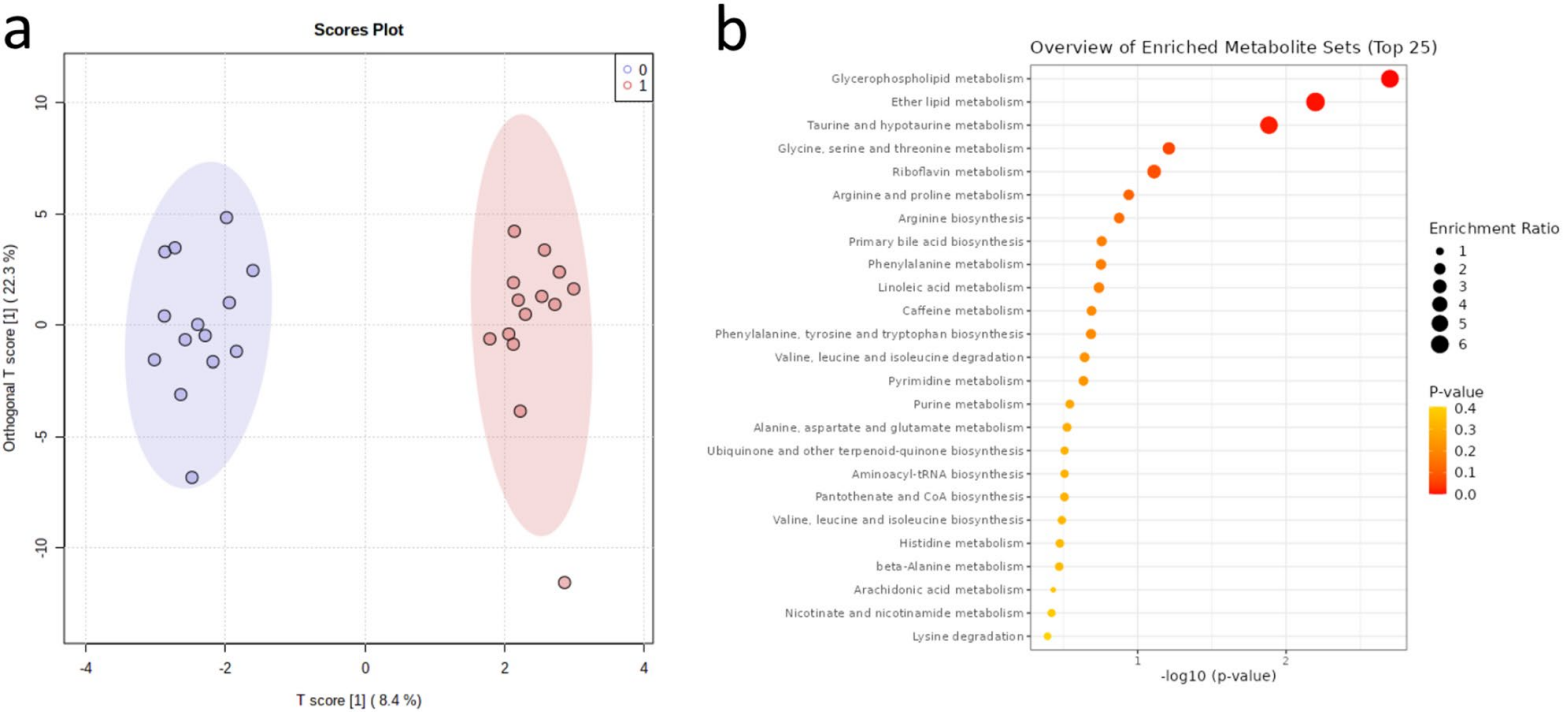
Multivariate metabolome analysis of the fecal samples. (**a**) Orthogonal partial least square discriminant analysis (OPLS-DA) of the fecal samples (n = 14), with the changes during the control period (0) and the exercise period (1). The model was validated with permutation tests (one hundred permutations p < 0.01) and cross-validation (R2Y = 0.918 and Q2 = 0.497). (**b**) KEGG metabolic pathways in the enrichment analysis of fecal samples in MetaboAnalyst. No metabolic pathways reached significance after correction for multiple tests.

We identified 154 metabolites in the fecal samples and ran multivariate analysis as for the serum samples. Fifty one metabolites of these were also detected in serum comprising largely of amino acids (18) and purines (5). The details for each fecal metabolite are listed in Supplementary Table S2. In OPLS-DA, 22.3% of the variation in the data was explained by the orthogonal component (Fig. 3a). Sixty metabolites of interest were selected using the thresholds p(corr)[1]  < − 0.2 or > 0.2 or VIP-values > 1.0, and the model was reassessed using only these metabolites. The cross-validation for the reduced model presented values of R^2^ = 0.918 and Q^2^ = 0.497, and the robustness of this model was measured by 100 permutations tests with p < 0.01. The most enriched pathways in the fecal metabolomes were glycerophospholipid, ether lipid, and taurine metabolic pathways (Fig. 3b). No pathways remained significant after correction for multiple tests. In disease signatures, no significant enrichments were found (Supplementary Fig. S4).

In univariate analysis, we found eleven fecal metabolites to alter during the study (Table 2), however, the p-values for fecal samples were not corrected for multiple testing. Glycerophosphocholine, proline betaine, histidinylproline, and inosine increased during the exercise period. Gamma-glutamyltyrosine, gamma-glutamylleucine, and alpha-linolenic acid increased whereas methylxanthine and proline betaine decreased during the control period. In addition, TML, 4-hydroxycyclohexyl-carboxylic acid, and 3-phenyllactic acid increased towards the mid-time point and decreased subsequently, with no observable changes during the control period.

**Table 2:** Mean abundances ± standard deviations of the fecal metabolites with significant changes during the study.

Compound	Class/ontology	Linear mixed model p-value	Pre	Post1 (n = 16)	Mid	Post2 (n = 15)
4-Hydroxycyclohexyl-carboxylic acid	Exposome	0.048	5.81 ± 0.41	5.91 ± 0.46	**5.99 ± 0.43**^**#**^	5.76 ± 0.31
Methylxanthine	Caffeine	0.070	5.5 ± 0.97	**5.32 ± 0.93**^**#**^	5.33 ± 0.99	5.5 ± 0.94
3-Phenyllactic acid	Amino acids	0.063	6.12 ± 0.67	6.13 ± 0.34	**6.52 ± 0.8**^#^*	6.43 ± 0.88
alpha-Linolenic acid	Fatty acid	0.085	6.36 ± 0.73	**6.82 ± 0.53**^**#**^	**6.78 ± 0.54**^**#**^	6.68 ± 0.56
gamma-Glutamylleucine	Amino acid	0.078	5.56 ± 0.74	**5.89 ± 0.3**^**#**^	**5.85 ± 0.36**^**#**^	5.82 ± 0.33
gamma-Glutamyltyrosine	Amino acid	0.098	5.48 ± 0.31	**5.63 ± 0.32**^**#**^	5.54 ± 0.4	5.53 ± 0.38
Glycerophosphocholine	Phospholipid	0.068	4.98 ± 0.46	4.87 ± 0.59	5.03 ± 0.5	5.29 ± 0.6**
Histidinylproline	Amino acid	0.034	6.01 ± 0.45	5.99 ± 0.53	**6.22 ± 0.5**^#^*	**6.28 ± 0.32**^#^*
Inosine	Purines	0.057	5.24 ± 0.71	5.26 ± 0.46	5.45 ± 0.54	**5.61 ± 0.67**^#^*
N6,N6,N6-Trimethyllysine	Amino acid	0.078	6.42 ± 0.18	6.48 ± 0.22	**6.58 ± 0.29**^**#**^	6.45 ± 0.28
Proline betaine	Amino acid	0.019	7.79 ± 0.49	**7.37 ± 0.46**^**##**^	7.58 ± 0.47	7.73 ± 0.44*

The values are log10-transformed LC–MS peak signals. Values in bold indicate a significant change from a previous timepoint. Change from post1 p-value *< 0.05. Change from pre p-value ^#^< 0.05.

*Pre* before the control period, *Post1* after the control period, *Mid* midpoint of the exercise period, *Post2* after the exercise period.

### Network analysis linked the phospholipid signature with the gut microbiome

**Figure 4:**
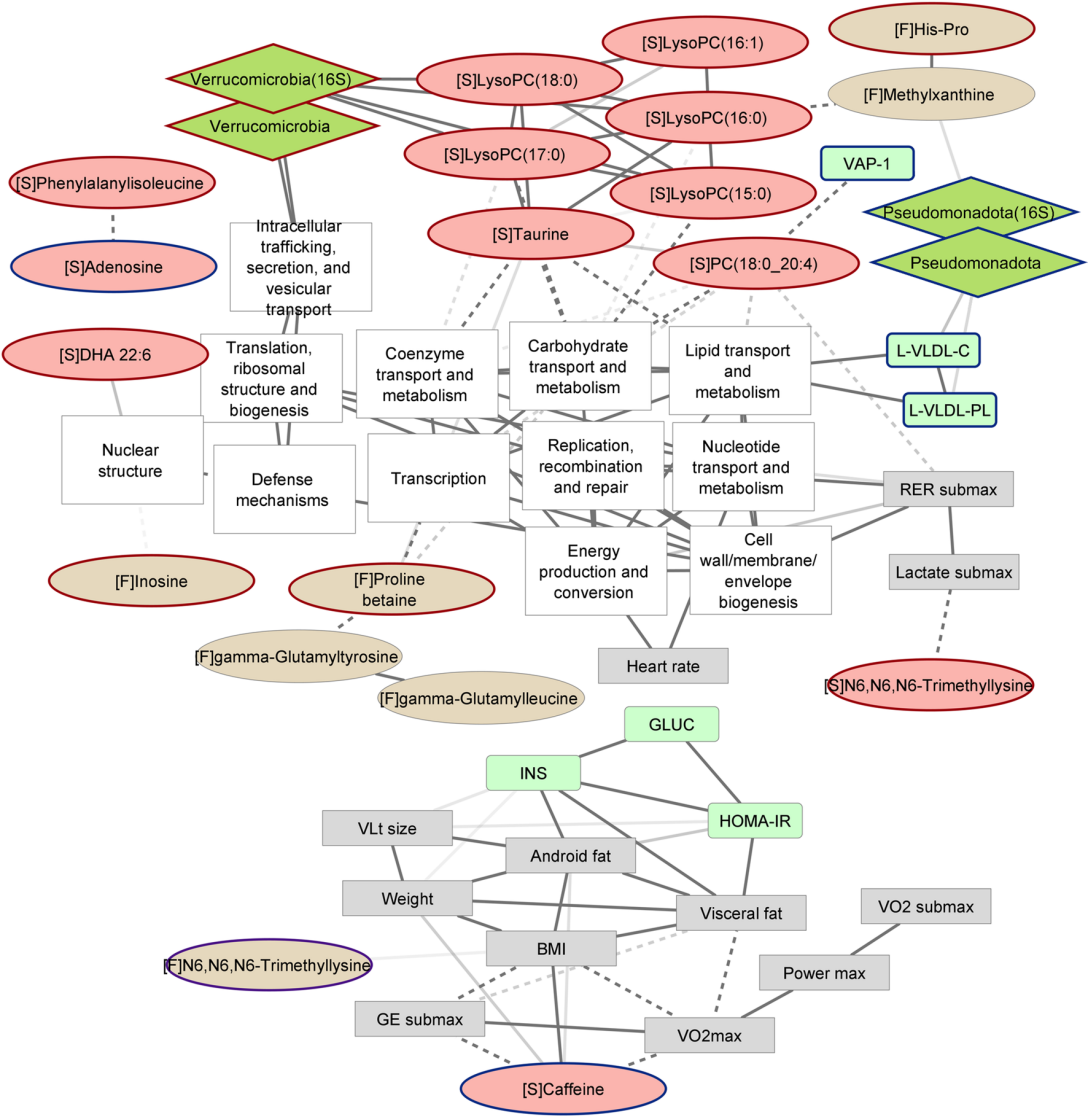
Correlation network between serum metabolites (red oval [S]), fecal metabolites (beige oval [F]), gut microbial taxa (green rhombus), gut microbial functions (white rectangle), biochemical markers (green rectangle) and functional variables (grey rectangle). Red node outline indicates a significant increase, blue outline indicates a significant decrease and purple outline indicates fluctuation during the exercise period. Solid line indicates a significant (Spearman p < 0.1 after FDR correction) positive correlation, dashed line indicates a significant negative correlation. The darker the edges, the smaller the p-values. Figure built with Cytoscape 3.

We pooled the samples from post1 and post2 timepoints, measured the Spearman correlations between the significantly altered metabolites and other variables and constructed a network of the significant associations. We then reduced the network using the Girvan-Newman algorithm, which is a hierarchical method for community discovery in complex systems. Compounds with a similar origin and related functional variables tended to cluster together, supporting the validity of the analysis used in this context. Serum lyso-phospholipids, along with taurine, were prominently cross-correlated and associated positively with the phylum Verrucomicrobiota (representing the genus *Akkermansia*). They associated inversely with microbial functions involving nutrient and coenzyme metabolism (Fig. 4). Serum PC(18:0_20:4) was inversely associated with the same pathways and inflammatory VAP-1 activity (semicarbazide-sensitive amine oxidase, SSAO). Serum caffeine associated positively with BMI and inversely with gross efficiency and peak oxygen uptake. Fecal proline betaine was associated positively with serum taurine and inversely with several microbial functions and fecal gamma-glutamyl amino acids. Fecal methylxanthine, a demethylation product of coffee-derived dimethylxanthines, correlated positively with histidinylproline and the phylum Pseudomonadota, and inversely with serum lysoPC(16:0). Lipids in large VLDLs associated positively with the phylum Pseudomonadota and microbial metabolic pathways in the metagenome involving carbohydrates and lipids, suggesting a connection between the gut microbiome and lipid metabolism. A biclustering analysis^21^ of the associations corroborated the links between phospholipids and microbial metabolism (Supplementary Fig. S1).

We used the same iteration of the network algorithm to search for communities within all the metabolites of interest, gut microbial taxa, and metabolic functions (Supplementary Fig. S2). Serum glycerophosphocholine and PCs formed a community and were associated inversely with fecal methylxanthine, the genus *Parabacteroides,* and the family *Porphyromonadaceae.* They associated positively with the genus *Akkermansia*, the family *Ruminococcaceae* and the family *Christensenellaceae*. Fecal leucine, phenylalanine, lysine, and histidinylproline clustered with several other dipeptides and amino acid derivatives and inversely correlated with *Methanobrevibacter.* Metabolites with similar origins tended to form clusters: Fecal PCs formed a community with choline cation and glycerophosphocholine, serum caffeine metabolites and coffee-derived compounds formed a community, and serum adenosine was inversely associated with the purine base xanthine.

## Discussion

We observed alterations in several metabolic pathways and serum metabolites in healthy women with overweight during an endurance training program. Several serum phospholipids, mainly lysoPCs, increased during the exercise period and covaried with several metabolic pathways in the gut metagenome. This co-occurred with an increase in the bacterial genus *Akkermansia*. Analyses of the fecal metabolome suggested alterations in glycerophospholipid metabolism during the exercise period and alterations in amino acid metabolism throughout the study.

Lysophospholipids can be generated from intact glycerophospholipids by phospholipase A1 and A2 and reactive oxygen species, which are both markers of increased inflammatory status^22^. Plasma lysoPC is additionally generated by lecithin cholesterol acyltransferase, which catalyzes the transacylation of fatty acid from membrane PC to free cholesterol in lipoprotein particles^23^. Mainly secreted by the liver, lysoPC is the most abundant lysophospholipid in the body and due to its inflammatory effects and its contribution to insulin signaling impairment is of particular interest within the lipidome^22, 24^. Pro-inflammatory actions such as the expression of adhesion molecules, release of chemotactic factors, and enhancing the production of reactive oxygen species have been attributed to saturated and monounsaturated lysoPCs such as lysoPC16:0 and lysoPC18:1^23^. Conversely, the polyunsaturated lysoPC species such as lysoPC(22:6) harbor anti-inflammatory properties, neutralizing the inflammatory effect induced by saturated lysoPC^23, 25^. The PC moieties containing odd-numbered acyl chains C15 and C17, which are less abundant in human tissues^26^, could either indicate microbial origin of fatty acids or altered propionyl-CoA availability^27^.

In our study the enzyme activity of the SSAO family member VAP-1 decreased as lysoPC species simultaneously increased. The activity also inversely correlated with the intact serum phospholipid PC(18:0_20:4), a source of stearyl and eicosatetranoyl moieties. LysoPC has been identified as an activator of human lung SSAO^28^, a slightly different member of the protein family, however PCs have not been linked to the homeostasis of VAP-1 -type amine oxidase^29^, at least so far. Both eicosanoids and VAP-1 are involved in inflammatory and cardiovascular processes^29–31^ thus an interaction is possible. Considering the simultaneous decrease in phospholipids in VLDL^18^, it is possible that increased physical activity enhances lipid oxidation and degradation of phospholipids in lipoproteins, having implications for cardiovascular health.

The gut microbial genus *Akkermansia* was found to increase during the intervention^18^ and in this study we report that it covaries with serum PCs (Fig. 4). The species *A. muciniphila* is the best-characterized representative of the phylum Verrucomicrobiota in the human gut and has raised interest for its potential health benefits, including, but not limited to, improved lipid oxidation and adaptive immune responses in the gut^32, 33^. This genus is a prominent degrader of the intestinal mucus layer and, although important for intestinal function, can also promote pathologic changes in certain conditions^34^. Although PCs serve as substrates to several gut microbes, their degradation or production has not been linked with *Akkermansia *per se in any previous publications. However, Gao et al*.* observed that a polyunsaturated glycerophosphatidylcholine supplementation could support *Akkermansia* population during high-fat diet-induced dysbiosis^35^. Tian et al*.* observed *Akkermansia* to correlate with fecal short chain fatty acids and several serum PCs^36^. Moreover, at least in obese animal models, *Akkermansia* seems to have a regulatory role in lipid metabolism. The genus has previously been shown to increase clearance of triglyceride-rich chylomicrons^37^, which could partly explain the observed decrease in VLDL-contained lipids. Increases in serum PCs could provide substrates for the gut microbiota*.* To support this assumption, we found the glycerophospholipid and ether lipid metabolic pathways altered in fecal samples and the fecal abundance of glycerophosphocholine to increase during the program. The lysoPCs that changed most during the intervention were inversely associated with coenzyme, carbohydrate, and lipid metabolic pathways in the gut metagenome. Large VLDLs are likely to be exercise-responsive lipoproteins^38–41^, but whether gut microbiome plays a role in the responses remains to be determined.

Related to lipid metabolism, we also found serum taurine levels to increase during the exercise program in a correlative fashion with serum PCs. Taurine associated with microbial lipid, carbohydrate, and coenzyme metabolic pathways. We also found the taurine and hypotaurine metabolic pathways enriched in the fecal samples. Taurine is a non-proteogenic aminosulfonic acid which is mainly obtained from dietary sources, but also synthesized in small amounts from methionine and cysteine^42^. Taurine has several functions in the body: it acts as an antioxidant and regulates energy metabolism in the skeletal muscle by inhibiting glycolysis and promoting fatty acid uptake for beta oxidation in the mitochondria^42^. In bile acid biosynthesis, taurine is conjugated with the primary bile acids by liver cells and excreted into the small intestine. The resulting bile salts can be metabolized by the microbiome in the large intestine into unconjugated primary and secondary bile acids^43, 44^. In the absence of significant dietary changes during our study^18^, the increase in serum taurine levels could be from improved absorption due to the action of the gut microbiota. Considering the many beneficial effects of maintaining taurine levels^42, 45^ it is of interest whether exercise can induce such changes.

Adenosine and inosine are intermediates in the degradation of purines and purine nucleosides to uric acid. In conditions where ATP hydrolysis outpaces the rate of ADP re-phosphorylation, such as during intense exercise or hypoxia, this degradation is upregulated^46^. Degradation products, such as purine nucleosides and bases, can be lost from muscle due to transport and/or diffusion across cell membranes. Purine bases hypoxanthine and xanthine are further oxidized into uric acid, the final product of purine metabolism, or salvaged through the action of hypoxanthine phosphoribyltransferase^47^. We found the concentrations of fecal inosine to increase and serum adenosine to drop during the exercise period, along with a covarying, non-significant, decrease in serum xanthine. This could indicate a shift towards adenosine nucleotide degradation, instead of salvage, prompted by increased physical activity.

Finally, serum caffeine increased during the control and subsequently decreased during the exercise period. We also observed inverse changes in fecal methylxanthine, and a related enrichment in the caffeine metabolism pathway without changes in caffeine intake of the participants during the study. Coffee-derived metabolites, such as caffeine and xanthines, are likely ubiquitous in Finnish populations who are the leading consumers of coffee per capita^48^ and therefore might be sensitive markers of health behavior changes. To support this, using the same metabolomics method, we recently identified serum caffeine and its main metabolite paraxanthine to be potential markers of fat content in the liver^20^. Notably, proline betaine, which we found to vary in feces, has also been identified as a marker of coffee^49^. Since caffeine degradation is dependent on hepatic enzyme activity, particularly of the cytochrome P40 family^50^, it is possible that an enhanced hepatic function in response to exercise^51^ is behind the alterations. Also, since caffeine is a major antagonist of adenosine receptors and could compete with adenosine for receptor binding^52^, it is of interest whether serum caffeine levels affect the flux of purines into circulation.

This study is not without limitations. The sample size is quite limited for omics analyses which can limit the applicability of findings. In particular for fecal metabolomics, our sample size was underpowered to confirm small to moderate effects because the concentrations of compounds in feces are highly fluctuant and depend on several uncontrollable factors such as sample water content and storage. This prompted us to focus mainly on prevalence and associations with other variables. A strength is that we analyzed the dietary components comprehensively and reported no changes in the dietary macronutrients as outlined in the previous publication^18^. The population in this study was limited to adult northern European women with overweight. This homogeneity is beneficial for studies on microbiome and fecal metabolome which are highly dependent on geographical location and dietary patterns although any application of the results to more diverse populations should be done carefully. The study design did not adhere to a typical controlled trial where a control group consists of separate individuals. Instead, we implemented a quasi-experimental design where two time points prior to the intervention were captured. This design is often preferable in microbial time-series studies due to the highly individual nature of the gut microbiome^53^. With metabolomics, although a randomized controlled design is often preferred, this quasi-experimental design allows us to better observe the effect of regression towards the mean^54^. Indeed, in univariate analysis we found some of the metabolites, particularly caffeine-related metabolites, fatty acids, TML and fecal gamma-amino acids to fluctuate over time regardless of whether programmed exercise was ongoing.

Previously sedentary women with overweight who underwent six weeks of endurance training improved their cardiorespiratory fitness and had shifts in their serum metabolome which did not occur during a preceding control period. In response to exercise, adenosine and caffeine decreased in serum, suggesting increases in purine nucleotide degradation in the skeletal muscle and caffeine metabolism in the liver. Most notably, serum taurine and PCs, particularly the lyso-moieties (Table 1), and fecal glycerophosphocholine (Table 2) showed an increase in response to aerobic exercise. This co-occurred with an increase in *Akkermansia,* a genus of bacteria essential to intestinal function^34^, and a decrease in phospholipids and cholesterol in L-VLDL particles. Absent of changes in dietary macronutrients or major changes in systemic metabolism, we suspect an exercise-induced increase in the degradation of PCs in lipoproteins and a gut microbial component involved in the process. This provides possible new insights into ways to induce beneficial changes in the gut microbiota and warrants further mechanistic investigation into phospholipid metabolism and exercise-responsive gut microbes, such as *A. muciniphila*.

## Methods

### Participants

Selection of the participants has been described in detail before^18^. Participants were recruited through advertisements in social media and a local newspaper having a circulation of approximately 70,000. Inclusion criteria were sedentary lifestyle and body mass index (BMI) > 27.5 kg/m^2^. Exclusion criteria were antibiotic treatment within 2 months, major inflammatory gastrointestinal disorders, major eating disorders, diagnosed type 1 or 2 diabetes mellitus, cardiovascular diseases other than hypertension, hypothyroidism or other endocrine disease that may affect training or the study outcomes, and musculoskeletal diseases that could preclude the ability to perform training and testing. Twenty female participants were initially enrolled into the study with 17 completing the exercise program and sampling (Table 3). The study was conducted in accordance with the Helsinki Declaration and approved by the ethical committee of the Central Finland Health Care district (KSSHP) (KSSHP document number 2U/2015). A written informed consent was obtained from all study participants before the study.

**Table 3:** Characteristics of the study sample (n = 17).

	Pre	Post1	Post2
Age (years)	36.8 ± 3.9	36.8 ± 3.9	36.8 ± 3.9
BP systolic (mmHg)	130 ± 12	131 ± 12	132 ± 12
BP diastolic (mmHg)	81 ± 11	82 ± 9	80 ± 6
Height (cm)	168 ± 6	168 ± 6	168 ± 6
Weight (kg)	90.1 ± 15.7	89.9 ± 15.0	89.3 ± 15.6
WC (cm)	98.7 ± 13.0	100.9 ± 11.7	99.3 ± 11
BMI (kg/m^2^)	31.8 ± 4.4	31.7 ± 4.2	31.4 ± 4.1

Numbers are mean ± standard deviation.

*BP* blood pressure, *WC* waist circumference, *BMI* body mass index, *Pre* before the control period, *Post1* after the control period, *Post2* after the exercise period.

### Exercise program and functional variables

The study design, collection of samples and measurements of functional variables have been described in detail before^18^. The study consisted of a six-week control period and a subsequent six-week exercise period (Fig. 1). Participants were asked to maintain individual habitual physical activity and eating habits throughout the study. Three training sessions were performed weekly. During weeks 1–2, 40 min steady-state cycling of low intensity was performed. During weeks 3–4, the duration of the exercise session was 50 min. Every other training session consisted of three 10-min intervals of moderate intensity cycling, with the rest of the training session performed at low intensity. Every other training session included only low intensity cycling. During weeks 5–6, the duration of training sessions was 60 min consisting of four 10-min intervals of moderate intensity cycling with the rest of the training session performed at low intensity. The training intensity was verified by heart rate, rate of perceived exertion and blood lactate measures in the beginning of the exercise period.

At the beginning (pre), middle point (post1) and end point (post2) of the study, fecal and serum samples were collected from the participants and diet was assessed using questionnaire. The participants were advised to maintain their habitual ad libitum diet. The intakes of total energy and energy-yielding nutrients were analyzed from self-reported 3-days food records (2 weekdays and 1 weekend day) using Micro-Nutrica software. Food records contained time of eating and the types and amounts of food and drink. The average daily intakes were calculated from the three days and used in the analyses. Cardiorespiratory fitness, body composition, maximal isometric force, and muscle thickness of vastus lateralis were measured at these time points. An additional fecal sample was collected at the midpoint of the exercise period for the untargeted metabolomics analysis (see below).

### Sample collection and processing

Collection and handling of serum and fecal samples was performed as reported before^18^. Briefly, blood samples were collected after an overnight fast, at least 72 h after the last exercise bout. Serum was separated by centrifuging at 3000×*g* for 10 min and stored at—− 80 °C until analysis. The participants collected the fecal samples at home, at least 72 h after the last exercise bout. Samples were frozen immediately at home freezers after collection, brought to laboratory frozen and stored at − 80 °C until processing.

### Metabolomics

For the extraction of serum metabolites, samples were thawed on ice and a 100 μL aliquot of plasma was dispensed into a 96-well filter plate (Captiva ND, 0.2 μm PP, Agilent Technologies) containing 400 μL of ice-cold acetonitrile. Samples were mixed to thoroughly precipitate plasma proteins, and then centrifuged 700×*g* for 5 min at 4 °C and the supernatants were collected to a 96-well storage plate and stored refrigerated. For the extraction of fecal metabolites, thawed samples were suspended in phosphate-buffered saline with a ratio of 1:5 (w:v) and vortexed for 10 min. An aliquot of 100 μL of the fecal slurry was mixed with 500 μL of ice-cold methanol on a 96-well filter plate (Captiva ND, 0.2 μm PP, Agilent Technologies). The plate was centrifuged at 700×*g* for 5 min at 4 °C and the supernatants were collected to a 96-well storage plate and stored refrigerated.

Nontargeted metabolic profiling was performed at the LC–MS metabolomics center (Biocenter Kuopio, University of Eastern Finland, Finland) as before^55^. The analysis was carried out using an ultra-high performance liquid chromatography (Vanquish Flex UHPLC system, Thermo Scientific, Bremen, Germany) coupled online to a high-resolution mass spectrometry (Q Exactive Focus, Thermo Scientific). All samples were analyzed using reversed phase (RP) and hydrophilic interaction chromatography (HILIC) techniques. Data were acquired in both positive and negative electrospray ionization (ESI) polarities. Data-dependent product ion spectrums (MS2 data) were acquired from pooled quality control (QC) samples at the beginning and end of the analysis for each mode. QC samples were injected in the beginning of the analysis and after every 12 samples. Peak detection and alignment was performed in MS-DIAL^55^ (version 4.9)^56^. Drift correction, normalization to quality control samples and clustering of molecular features was performed using the Notame^56^ (version 0.0.10) package in R (version 4.1)^57^. Features were flagged for low detection (< 80% prevalence in QC samples) and, subsequently, for low quality using the default cutoff values for coefficient of variation within the QC samples and the D-ratio^58^ between QC and biological samples. Flagged features were removed prior to clustering. Euclidean distance between QC samples were used to confirm run quality and principal component analysis (PCA) was used to check for outliers and clusters **(**Supplementary Fig. S3). Compounds were identified by comparing the mass spectra and retention times to an in-house mass spectrum library^59^. Secondarily, spectra were compared to publicly available references, and in-silico generated spectra using MS-FINDER (version 3.5). The details for the identified metabolites are listed in the supplements (Supplementary Tables S1 and S2).

### Gut microbiome and biochemical analyses

The methods to analyze serum lipid subclasses and VAP-1 activity (assessed by activity of SSAO) have been described in detail before^18^. For the gut microbiome, total bacterial DNA was extracted using GXT stool kit and semi-automated GenoXtract machine (Hain Lifescience, Nehren, Germany) accompanied with bead-beating. In 16S rRNA gene amplicon sequencing, the V4 region of the bacterial 16S rRNA gene was amplified. Then, the 16S rRNA gene libraries were sequenced with 2 × 250 bp paired-end reads on Illumina MiSeq system (Illumina, Inc. San Diego, ca-USA) using MiSeq v3 reagent kit (Illumina, Inc.). Regarding the taxonomic data, all analyses were made with QIIME1^60^ (version 1.9) from the randomly subsampled OTU table with rarefaction level matching the sample with the lowest total OTU count.

For the metagenomes, the DNA libraries were generated following Nextera XT Illumina protocol (#FC-131-1024, Illumina, San Diego, CA, USA) and 0.2 ng/μl of purified gDNA. The multiplexing step was performed using Nextera XT Index Kit (#FC-131-1096). The libraries were sequenced using 2 × 300 pb paired-end run (#MiSeq Reagent kit v3 #MS-102-3001). Reads containing ribosomal gene fragments were passed to taxonomic analysis, and taxonomic annotation was carried out with SILVA Incremental Aligner (SINA) v1.2.10 using SILVA Release 123.1. The rest of the reads were used for open reading frames (ORFs). The database of Clusters of Orthologous Groups (COGs) was used to identify the predicted genes and their relative abundance. The COGs database contained 4631 orthologous proteins based on the annotation of 711 microbial genomes that represent the diversity of bacteria and archaea. All predicted proteins from the fecal samples were mapped onto the COGs database via BLASTP searches using a cut-off of 10^−10^ and selection of the best blast hit. The functional annotation of all ORFs was performed in two steps: (1) a BLASTP search using an e-value cut-off of 10^−10^ to filter out random matches and (2) selection of only one matching sequence based on the best blast hit to prevent cross-reference among genes.

### Statistical analyses

We performed multivariate analysis of the metabolites using MetaboAnalyst (version 5). Briefly, we calculated the fold changes for each metabolite during control or exercise period and applied log-transformation and autoscaling to the fold change values. We applied OPLS-DA to verify the differences between the control and the exercise periods. We used the S-plot and VIP-plot to identify metabolites with the most contribution to the differences. Following a visual inspection of the S-plot and the related VIP values, we selected metabolites of interest based on the p(corr)[1] and VIP values. We assessed the robustness and quality of the model by permutation tests (100 permutations) and cross-validation (R2Y and Q2). Subsequently, the fold changes were subjected to enrichment analysis in MetaboAnalyst to evaluate for aberrations in Kyoto Encyclopedia of Genes and Genomes (KEGG) metabolic pathways or disease signatures^61^.

We conducted univariate and association analyses using R (version 4.1). A log-transformation was applied and univariate analyses on the selected metabolites were used to test for significant changes. For serum metabolites, first, the data distribution and homogeneity were tested using the Shapiro–Wilk test. Depending on the distribution, either one-way repeated measures ANOVA or the Friedman test was used to test for significant differences between the time points and the p-values were corrected (q-values) for multiple tests using the Benjamini–Hochberg false discovery rate (FDR). Post hoc tests, either t-test or Wilcoxon, were conducted on significant metabolites (ANOVA/Friedman q-value < 0.1) with corrections for multiple comparisons using the Holm method. For fecal metabolites, to mitigate missing samples in the post1 and post2 time points, a linear mixed model (package lme4, version 1.1) was utilized instead of ANOVA. For each metabolite as a dependent variable, the model was fitted with time point as a fixed effect and participant as a random effect. A post hoc test of estimated marginal means for pairwise comparisons (package emmeans, version 1.8) was conducted for each linear model with p-value < 0.1. Due to low power in the fecal data, no multiple test corrections were utilized.

To explore associations with the metabolomes, the gut microbiome, and physiological effects of the exercise program, we combined the samples from post1 and post2 time points and ran Spearman correlations between the metabolites, significantly changed microbial taxa (Verrucomicrobiota and Pseudomonadota), microbial metabolic pathways and functional variables. Prior to the analysis, the metabolite abundances were log-transformed. The bacterial sequence counts were filtered for low prevalence (< 10%) taxa and transformed using the center log ratio. All the p-values were adjusted using false discovery rate and a network graph was constructed using the variables as nodes and the significant correlations (Spearman p fdr < 0.1a) as edges. We then ran the Girvan-Newman algorithm on the graph retaining only the edges with the highest betweenness-centrality using python 3 and the package NetworkX (version 2.6). We then visualized the network using Cytoscape 3 (version 3.9). In addition, we also used biclustering (Python 3, package Scikit learn 1.1), as documented before^21^, on the spearman correlations between metabolites and microbial metabolic functions to search for biologically relevant groups.

We conducted post-hoc power analysis for multivariate data using Metaboanalyst. Assuming a false discovery rate (FDR) of 0.2 and a mean effect size estimated from all the metabolites retained after filtering by OPLS-DA, we estimated statistical powers of at least 80% and 20% for paired comparisons of serum and fecal metabolites, respectively.

## Supplementary Information

Supplementary Information.

## Data Availability

The access to the data is restricted due to personal information protection (General Data Protection Regulation (GDPR) 2016/679 and Directive 95/46/EC). However, the datasets used in the current study are available from the corresponding author on reasonable request.

## References

[1] Reiner M, Niermann C, Jekauc D, Woll A (2013). Long-term health benefits of physical activity—A systematic review of longitudinal studies. BMC Public Health.

[2] Schroeder, E. C., Franke, W. D., Sharp, R. L. & Lee, D. C. Comparative effectiveness of aerobic, resistance, and combined training on cardiovascular disease risk factors: A randomized controlled trial. *PLoS One* **14**, e0210292 (2019). 10.1371/journal.pone.0210292 PMC6322789 30615666

[3] Hargreaves M, Spriet LL (2020). Skeletal muscle energy metabolism during exercise. Nat. Metab..

[4] de Feo P (2003). Metabolic response to exercise. J. Endocrinol. Invest..

[5] Fiuza-Luces C (2018). Exercise benefits in cardiovascular disease: Beyond attenuation of traditional risk factors. Nat. Rev. Cardiol..

[6] Karstoft K, Pedersen BK (2016). Exercise and type 2 diabetes: Focus on metabolism and inflammation. Immunol. Cell Biol..

[7] Clauss M, Gérard P, Mosca A, Leclerc M (2021). Interplay between exercise and gut microbiome in the context of human health and performance. Front. Nutr..

[8] Aya V, Flórez A, Perez L, Ramírez JD (2021). Association between physical activity and changes in intestinal microbiota composition: A systematic review. PLoS ONE.

[9] Campaniello D (2022). How diet and physical activity modulate gut microbiota: Evidence, and perspectives. Nutrients.

[10] Lensu S, Pekkala S (2021). Gut microbiota, microbial metabolites and human physical performance. Metabolites.

[11] Koh A, Bäckhed F (2020). From association to causality: The role of the gut microbiota and its functional products on host metabolism. Mol. Cell.

[12] Dohnalová L (2022). A microbiome-dependent gut–brain pathway regulates motivation for exercise. Nature.

[13] Sakaguchi CA, Nieman DC, Signini EF, Abreu RM, Catai AM (2019). Metabolomics-based studies assessing exercise-induced alterations of the human metabolome: A systematic review. Metabolites.

[14] Fiehn O (2002). Metabolomics—the link between genotypes and phenotypes. Plant Mol. Biol..

[15] Kelly, R. S., Kelly, M. P. & Kelly, P. Metabolomics, physical activity, exercise and health: A review of the current evidence. *Biochim. Biophys. Acta (BBA) Mol. Basis Dis.* **1866**, 165936 (2020). 10.1016/j.bbadis.2020.165936 PMC7680392 32827647

[16] Schranner D, Kastenmüller G, Schönfelder M, Römisch-Margl W, Wackerhage H (2020). Metabolite concentration changes in humans after a bout of exercise: A systematic review of exercise metabolomics studies. Sports Med. Open.

[17] Zierer J (2018). The fecal metabolome as a functional readout of the gut microbiome. Nat. Genet..

[18] Munukka E (2018). Six-week endurance exercise alters gut metagenome that is not reflected in systemic metabolism in over-weight women. Front. Microbiol..

[19] Cani PD, de Vos WM (2017). Next-generation beneficial microbes: The case of Akkermansia muciniphila. Front. Microbiol..

[20] Driuchina, A. *et al.* Identification of gut microbial lysine and histidine degradation and CYP-dependent metabolites as biomarkers of fatty liver disease. *mBio* **14**, e02663–22 (2023). 10.1128/mbio.02663-22 PMC9973343 36715540

[21] Hintikka J (2021). Xylo-oligosaccharides in prevention of hepatic steatosis and adipose tissue inflammation: Associating taxonomic and metabolomic patterns in fecal microbiomes with biclustering. Int. J. Environ. Res. Public Health.

[22] Mastrangelo A (2016). Insulin resistance in prepubertal obese children correlates with sex-dependent early onset metabolomic alterations. Int. J. Obes..

[23] Tan ST, Ramesh T, Toh XR, Nguyen LN (2020). Emerging roles of lysophospholipids in health and disease. Prog. Lipid Res..

[24] Han MS (2011). Lysophosphatidylcholine as an effector of fatty acid-induced insulin resistance. J. Lipid Res..

[25] Huang LS, Hung ND, Sok DE, Kim MR (2010). Lysophosphatidylcholine containing docosahexaenoic acid at the sn-1 position is anti-inflammatory. Lipids.

[26] Jenkins B, West JA, Koulman A (2015). A review of odd-chain fatty acid metabolism and the role of pentadecanoic acid (C15:0) and heptadecanoic acid (C17:0) in health and disease. Molecules.

[27] Qin, N., Li, L., Wang, Z. & Shi, S. Microbial production of odd-chain fatty acids. *Biotechnol. Bioeng.* **120**, (2023). 10.1002/bit.28308 36522132

[28] Dalfó, E., Hernandez, M., Lizcano, J. M., Tipton, K. F. & Unzeta, M. Activation of human lung semicarbazide sensitive amine oxidase by a low molecular weight component present in human plasma. *Biochim. Biophys. Acta (BBA) Mol. Basis Dis.* **1638**, 278–286 (2003). 10.1016/s0925-4439(03)00094-2 12878330

[29] Salmi M, Jalkanen S (2019). Vascular adhesion protein-1: A cell surface amine oxidase in translation. Antioxid. Redox. Signal.

[30] Danielli M, Thomas RC, Quinn LM, Tan BK (2022). Vascular adhesion protein-1 (VAP-1) in vascular inflammatory diseases. VASA.

[31] Sonnweber T, Pizzini A, Nairz M, Weiss G, Tancevski I (2018). Arachidonic acid metabolites in cardiovascular and metabolic diseases. Int. J. Mol. Sci..

[32] Everard A (2013). Cross-talk between Akkermansia muciniphila and intestinal epithelium controls diet-induced obesity. Proc. Natl. Acad. Sci. USA.

[33] Bae M (2022). Akkermansia muciniphila phospholipid induces homeostatic immune responses. Nature.

[34] Luo, Y. *et al.* Rational consideration of *Akkermansia muciniphila* targeting intestinal health: advantages and challenges. *NPJ Biofilms Microbiomes ***8**, 1–11 (2022). 10.1038/s41522-022-00338-4 PMC9576740 36253412

[35] Gao X (2021). Effect of different phosphatidylcholines on high fat diet-induced insulin resistance in mice. Food Funct..

[36] Tian T (2022). Multi-omics data reveals the disturbance of glycerophospholipid metabolism caused by disordered gut microbiota in depressed mice. J. Adv. Res..

[37] Xu Y (2020). Function of Akkermansia muciniphila in obesity: Interactions with lipid metabolism, immune response and gut systems. Front. Microbiol..

[38] Lehtovirta M (2022). Association of physical activity with metabolic profile from adolescence to adulthood. Scand. J. Med. Sci. Sports.

[39] Kujala UM (2013). Long-term leisure-time physical activity and serum metabolome. Circulation.

[40] Bell, J. A. *et al.* Associations of device-measured physical activity across adolescence with metabolic traits: Prospective cohort study. *PLoS Med.* **15**, (2018). 10.1371/journal.pmed.1002649 PMC6133272 30204755

[41] Jones PR (2019). Associations of physical activity and sedentary time with lipoprotein subclasses in Norwegian schoolchildren: The Active Smarter Kids (ASK) study. Atherosclerosis.

[42] Seidel U, Huebbe P, Rimbach G (2019). Taurine: A regulator of cellular redox homeostasis and skeletal muscle function. Mol. Nutr. Food Res..

[43] Jia W, Xie G, Jia W (2017). Bile acid–microbiota crosstalk in gastrointestinal inflammation and carcinogenesis. Nat. Rev. Gastroenterol. Hepatol..

[44] Schoeler M, Caesar R (2019). Dietary lipids, gut microbiota and lipid metabolism. Rev. Endocr. Metab. Disord..

[45] Staels B, Fonseca VA (2009). Bile acids and metabolic regulation: Mechanisms and clinical responses to bile acid sequestration. Diabetes Care.

[46] Miller SG, Hafen PS, Brault JJ (2019). Increased adenine nucleotide degradation in skeletal muscle atrophy. Int. J. Mol. Sci..

[47] Mikami T, Kitagawa J (2006). Intense exercise induces the degradation of adenine nucleotide and purine nucleotide synthesis via de novo pathway in the rat liver. Eur. J. Appl. Physiol..

[48] Usva K, Sinkko T, Silvenius F, Riipi I, Heusala H (2020). Carbon and water footprint of coffee consumed in Finland—life cycle assessment. Int. J. Life Cycle Assess..

[49] Papandreou C (2019). Plasma metabolites associated with coffee consumption: A metabolomic approach within the PREDIMED study. Nutrients.

[50] Hakooz N (2009). Caffeine metabolic ratios for the in vivo evaluation of CYP1A2, N-acetyltransferase 2, xanthine oxidase and CYP2A6 enzymatic activities. Curr. Drug Metab..

[51] Knudsen JG, Bertholdt L, Gudiksen A, Gerbal-Chaloin S, Rasmussen MK (2018). Skeletal muscle interleukin-6 regulates hepatic cytochrome P450 expression: Effects of 16-week high-fat diet and exercise. Toxicol. Sci..

[52] Ribeiro JA, Sebastio AM (2010). Caffeine and adenosine. J. Alzheimer’s Dis..

[53] Qian XB (2020). A guide to human microbiome research: Study design, sample collection, and bioinformatics analysis. Chin. Med. J. (Engl).

[54] Barnett AG, van der Pols JC, Dobson AJ (2005). Regression to the mean: What it is and how to deal with it. Int. J. Epidemiol..

[55] Lapatto, H. A. K. *et al.* Nicotinamide riboside improves muscle mitochondrial biogenesis, satellite cell differentiation, and gut microbiota in a twin study. *Sci. Adv.* **9**, (2023). 10.1126/sciadv.add5163 PMC9839336 36638183

[56] Tsugawa H (2015). MS-DIAL: Data-independent MS/MS deconvolution for comprehensive metabolome analysis. Nat. Methods.

[57] Klåvus A (2020). “Notame”: Workflow for non-targeted LC–MS metabolic profiling. Metabolites.

[58] Broadhurst D (2018). Guidelines and considerations for the use of system suitability and quality control samples in mass spectrometry assays applied in untargeted clinical metabolomic studies. Metabolomics.

[59] Schymanski EL (2014). Identifying small molecules via high resolution mass spectrometry: Communicating confidence. Environ. Sci. Technol..

[60] Caporaso, J. G. *et al.* QIIME allows analysis of high-throughput community sequencing data. *Nat. Methods* **7**, 335–336 (2010). 10.1038/nmeth.f.303 PMC3156573 20383131

[61] Kanehisa M, Furumichi M, Sato Y, Kawashima M, Ishiguro-Watanabe M (2023). KEGG for taxonomy-based analysis of pathways and genomes. Nucleic Acids Res..

