# Supplementary material for: Aerobic exercise training and gut microbiome-associated metabolic shifts in women with overweight: a multi-omic study
Source: Sci Rep. 2023 Jul 11;13:11228. doi: 10.1038/s41598-023-38357-6 (PMC10336137; doi:10.1038/s41598-023-38357-6)
Supplement: Supplementary file 1 — Supplementary Information. [file 41598_2023_38357_MOESM1_ESM.zip › suomiliikunta_supplements_rev.pdf]

Supplementary files

Hintikka, J *et al*. Aerobic exercise training and gut microbiome-associated metabolic shifts in women with overweight: A multi-omic study. 2023.

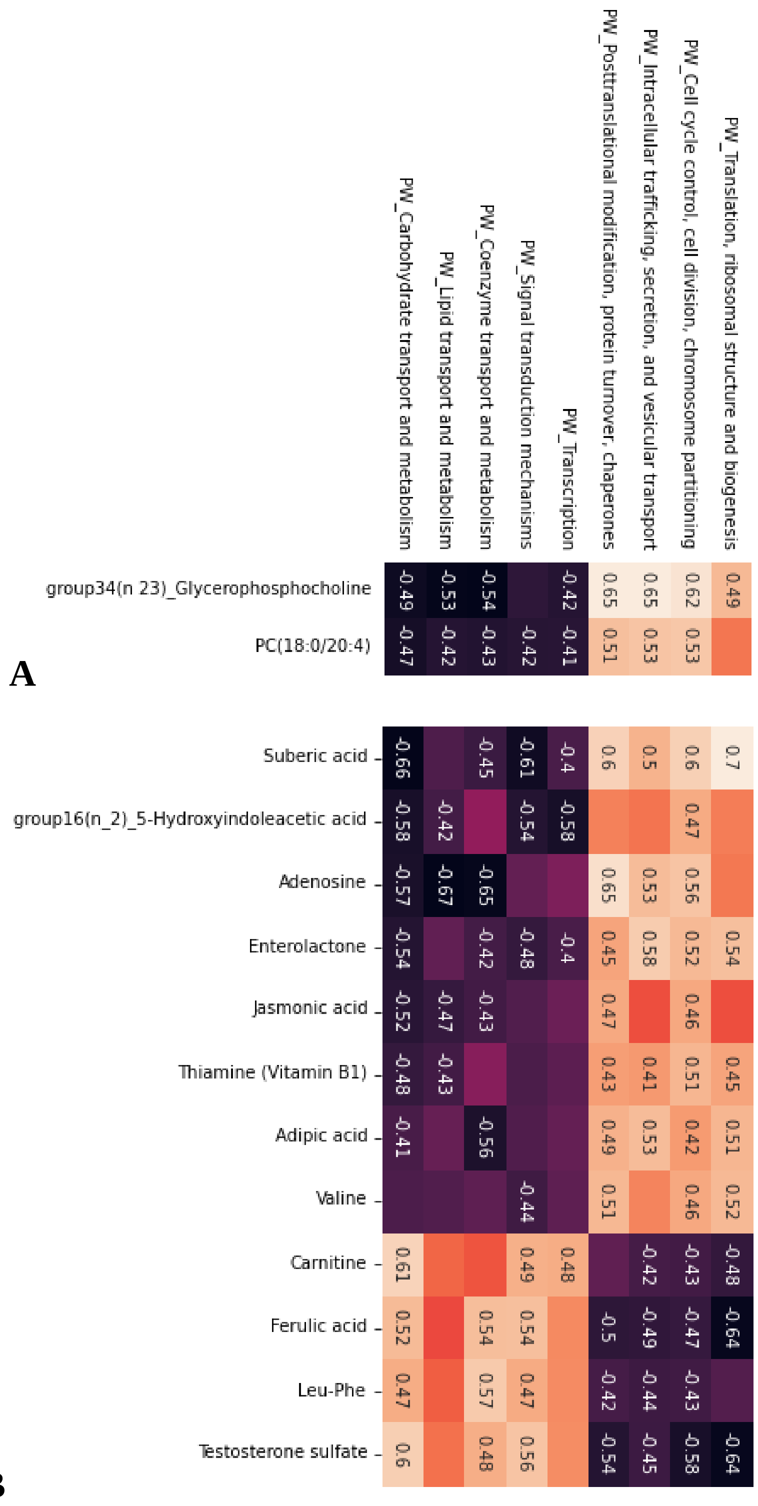

Figure S1*. Spearman correlations of gut bacterial functions with* ***A)*** *serum metabolites and* ***B)*** *fecal metabolites. Biclusters of functions and metabolites found using spectral biclustering. Serum group 34 corresponds to phospholipids and lysophospholipids. Feces group 16 corresponds to hydroxyindoleacetic acid and hippuric acid*

Supplementary files

Hintikka, J *et al*. Aerobic exercise training and gut microbiome-associated metabolic shifts in women with overweight: A multi-omic study. 2023.

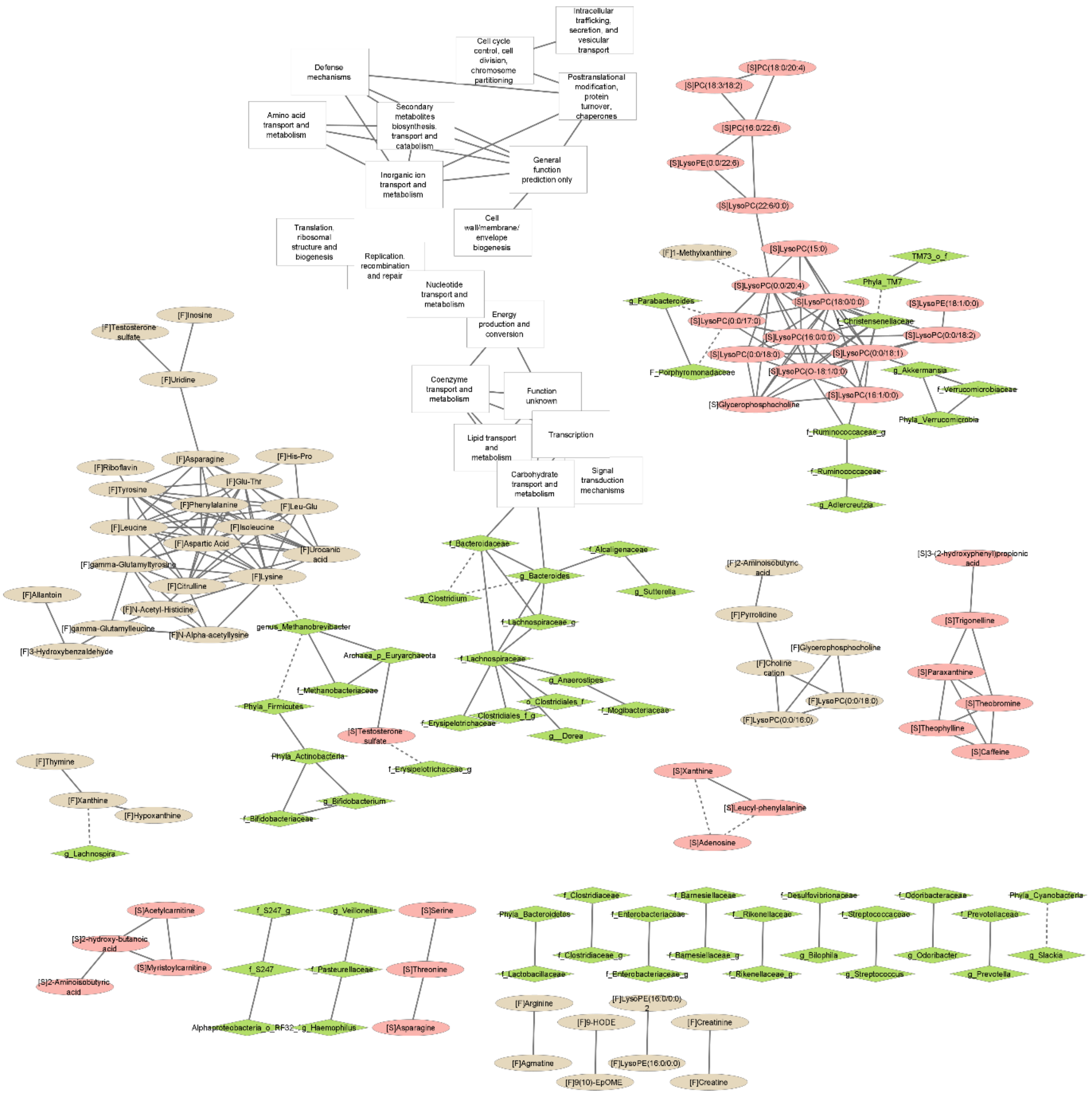

Figure S2. *Correlations between serum metabolites (red sphere [S]), fecal metabolites (beige sphere [F]), gut microbial taxa (green diamond) and gut microbial functions (white rectangle). Solid line indicates a significant (Spearman p <0.05 after Bonferroni correction) positive correlation, dashed line indicates a significant negative correlation. Communities analyzed using the Girvan-Newman algorithm in Python. Figure built with Cytoscape 3.*

Supplementary files
Hintikka, J *et al*. Aerobic exercise training and gut microbiome-associated metabolic shifts in women with overweight: A multi-omic study. 2023.

B

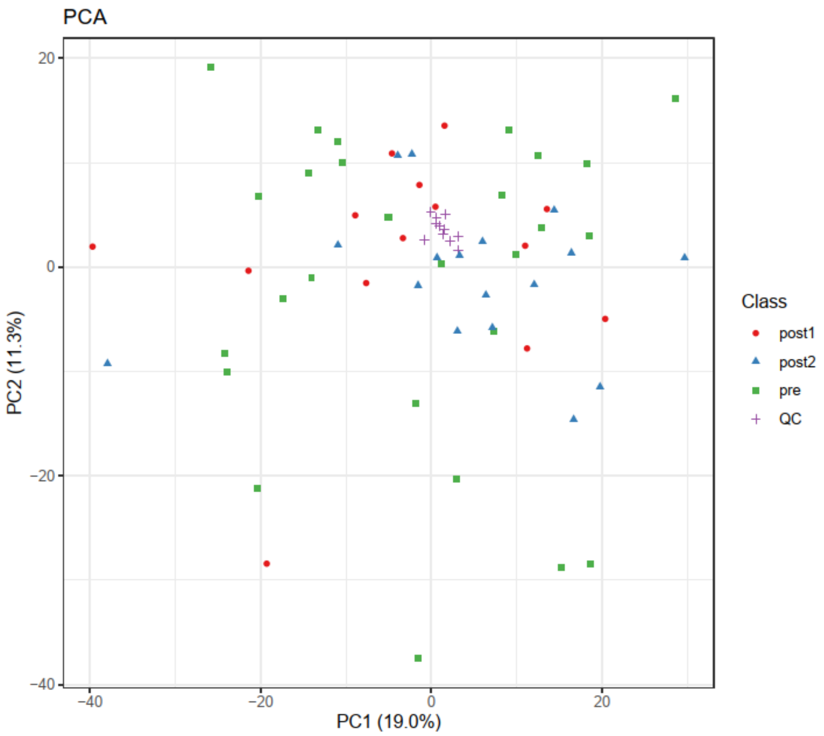

A

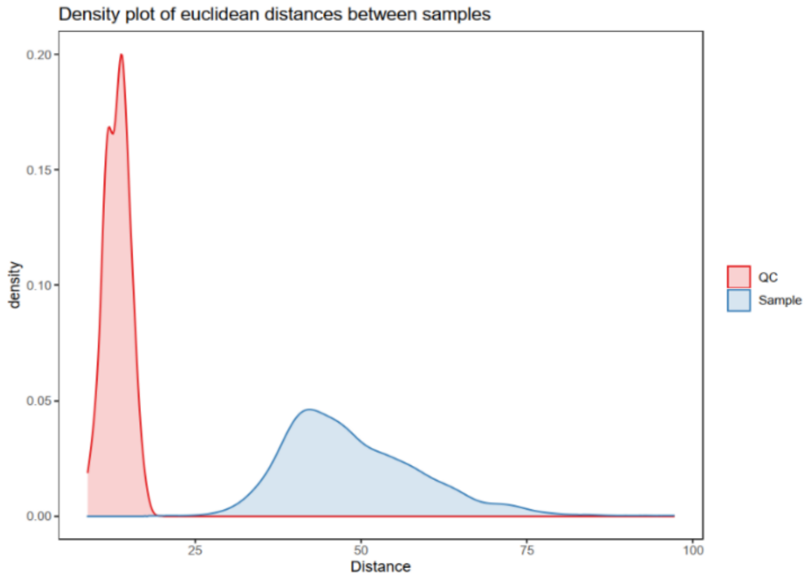

Figure S3. A) *Principal component analysis (PCA) plot and* ***B)*** *density plot of the serum samples from UHPLC-HRMS run in positive electrospray ionization, using a reverse phase column. PCA and the euclidean distances show close clustering of the quality control (QC) samples after drift correction.*

Supplementary files
Hintikka, J *et al*. Aerobic exercise training and gut microbiome-associated metabolic shifts in women with overweight: A multi-omic study. 2023.

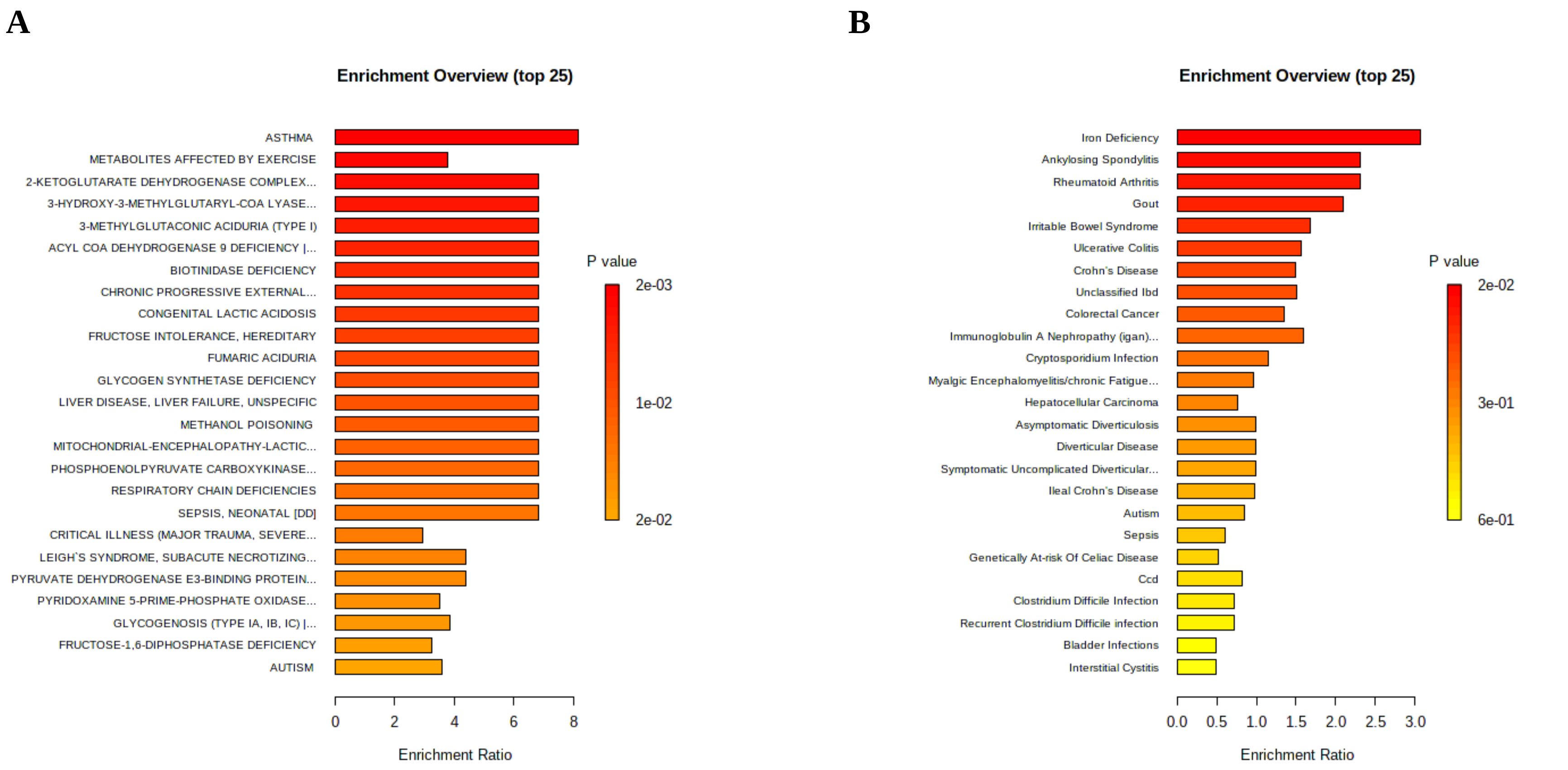

Figure S4. Disease signatures in the enrichment analysis of A) serum and B) fecal samples in MetaboAnalyst. No metabolic pathways reached significance after correction for multiple tests.
